# The Emotional Effectiveness of Advertisement

**DOI:** 10.3389/fpsyg.2020.02088

**Published:** 2020-09-04

**Authors:** F. Javier Otamendi, Dolores Lucia Sutil Martín

**Affiliations:** Brain Research Lab, Universidad Rey Juan Carlos, Madrid, Spain

**Keywords:** spots, target groups, emotions, valence, engagement, attention, facial expressions

## Abstract

Based on cognitive–emotional neuroscience, the effectiveness of advertisement is measured in terms of individuals’ unconscious emotional responses. Using AFFDEX to record and analyze facial expressions, a combination of indicators that track both basic emotions and individual involvement is used to quantitatively determine if a spot causes high levels of ad liking in terms of attention, engagement, valence, and joy. We use as a test case a real campaign, in which a spot composed of 31 scenes (images, text, and the brand logo) is shown to subjects divided into five groups in terms of age and gender. The target group of mature women shows statistically more positive emotions and involvement than the rest of the groups, demonstrating the emotional effectiveness of the spot. Each other experimental groups show specific negative emotions as a function of their age and for certain blocks of scenes.

## Introduction

Measuring the effectiveness of an advertising campaign is a major challenge for most companies, marketing professionals, and scientists in the twenty-first century (McCarthy and McDaniel, 2000). Starting back in the 1960s, a first conceptual model was developed to define the effectiveness of advertising. The “Hierarchy of Effects” model (Lavidge and Steiner, 1961) articulated customer response to spots in three stages: (1) cognitive, characterized by consciousness and information gathering, (2) affective, where liking for the spot and preferences for the product are set, and (3) behavior, where propensity or actual buy takes place. Following this model, the measurement of the impact of the consumer’s response to advertising campaigns was carried out through studies primarily aimed at the conscious cognitive processes of consumers (self-reporting, surveys, focus groups, etc.; Lewinski et al., 2014). These approaches generally failed to provide clear findings because they were not able to observe what happens in the consumer’s brain while taking a decision, they did not understand the role that emotions play both in understanding the message and in decision making, and they did not fully capture the way in which consumers process and understand cognitive responses to messages in advertising (Morin, 2011; Bercea, 2012; Cherubino et al., 2019). Moreover, in most cases, these techniques generated strong biases, such as social desirability (Benstead, 2013).

A new discipline, called consumer neuroscience, was born to try to resolve these voids of the previous model, going beyond emphasizing the conscious cognitive processes. Consumer neuroscience based “its objective in better understanding the consumer, through their unconscious processes. Explaining consumer preferences, motivations and expectations, predicting their behavior, and explaining successes or failures of advertising messages” (Bercea, 2012).

Consumer neuroscience was developed to penetrate in the consumers’ brain, and one of its focuses is on measuring effectiveness of advertising more precisely. The focus was shifted from the cognitive processes (stage 1 of the “Hierarchy of Effects” model), which were no longer considered to be the main drivers of consumer behavior, toward emotions and sentiments (primarily stage 2), which incorporated perceptions, experience, and recall (Halls, 2002). More precisely, marketing professionals and researchers, when measuring emotional responses following consumer neuroscience principles, were able to evaluate the unconscious assessment of the respondent (Poels and Dewitte, 2006; Lewinski et al., 2014; Varan et al., 2015), thereby providing a greater understanding of the effects of emotion on memory (Vecchiato et al., 2013).

Since the turn of the century, emotions have therefore been proposed to be a good predictor of advertising effectiveness (Poels and Dewitte, 2006) with a known important impact also in the cognitive process (Hamelin et al., 2017). Moreover, emotions have demonstrated to be necessary for the human function because they are strongly correlated with attention, decision-making, and memory (Le Blanc et al., 2014). Emotions also had an impact on the allocation of resources to the visual system and that more attention is placed on negative than on neutral stimuli (Öhman and Mineka, 2001; Algom et al., 2004; Estes and Verges, 2008).

Emotions have also been shown to impact highly on an individual’s response to receiving a message (Mai and Schoeller, 2009; Lewinski et al., 2014). Likewise, providing an emotional message in publicity increases the audience’s attention to the advertisement, and the product enhances the product’s appeal and generates a higher level of brand recall. Indeed, advertisements with emotional content are more likely to be remembered than those conveying news (Page et al., 1990).

Therefore, one necessary approach in this day and age to quantify the effectiveness of advertisements is to resort to emotions and emotional responses in the quest for properly measuring “ad liking and purchase intent” (McDuff et al., 2015).

To successfully achieve this quantitative challenging task, the pillars of consumer neuroscience are cognitive–emotional neuroscience, neuroimaging technologies, and biometric measurements, which together allow for obtaining objective data about emotions after observing and studying in real time what happens inside the consumer’s brain. The available tools permit the analysis of emotional activity without cognitive biases, providing instantaneous and continuous data that can be broken down into small pieces of study.

Accordingly, both advertising and marketing companies look for new or improved models, methodologies, indicators, tools, and techniques that can evaluate and predict consumer behavior based on unconscious emotional responses, making it difficult for customers to hide their true response. Some of these tools focuses on recording in a real environment or using virtual reality (VR) the metabolic activity [functional magnetic resonance imaging (fMRI), positron emission tomography], others on recording electrical activity in the brain [electroencephalography (EEG), magnetoencephalography, transcranial magnetic stimulation, steady-state topography], and still others without recording brain activity [eye tracking (ET), galvanic skin response (GSR), electromyography (EMG) or facial expression recognition].

These neuroscience tools are becoming popular for quantifying the emotional effectiveness advertising, especially (1) ET (Wedel and Pieters, 2008; Ramsøy et al., 2012; Lewinski et al., 2014; De Oliveira et al., 2015), (2) analysis of facial microexpression (Teixeira et al., 2012; Lewinski et al., 2014; Wedel and Pieters, 2014; Taggart et al., 2016), (3) fMRI (Bakalash and Riemer, 2013; Venkatraman et al., 2015; Couwenberg et al., 2017), and (4) VR (Bigné et al., 2016).

As for the analysis of facial microexpressions, different software was used to assess the effectiveness of advertising using the Facial Action Coding System, for example, FaceReader-FEBE system (Lewinski et al., 2014), GfK-EMO Scan software (Hamelin et al., 2017), and FACET and AFFDEX (Stöckli et al., 2018).

In all these studies, the measurement of emotions was mainly focused on understanding the seven basic emotions proposed by Ekman (1972): two positive (joy and surprise) and five negative (anger, contempt, disgust, fear, and sadness). In some cases, valence was also analyzed, directly from facial expressions or coupled with questionnaires (Timme and Brand, 2020).

It is important that all the available information is used to enrich the studies. On that regard, AFFDEX is a state-of-the-art software that, after recording people’s faces in front of stimuli, provides not only seven indicators about the likelihood on the emotional response being present in terms of the Ekman’s basic emotions, but also three additional indicators about the emotional involvement of the individual, namely, attention (focus of the participants during the experiment based on head position), engagement (the emotional responsiveness that the stimuli trigger), and valence (the positive or negative nature of their experimental experience; iMotions, 2020). All of the seven emotional indicators are scored in a normalized scale indicating the proportional likelihood of detecting the emotion. Attention and engagement have a range from 0 to 100, whereas the range of valence is from −100 to 100, providing an indication of positive, neutral, or negative experience. The initial thresholds are usually arbitrarily set at ±50 (iMotions, 2020).

Its applications in the last few years in diverse fields are numerous, for example:

- geriatric care (Taggart et al., 2016),
- forensics (Lei et al., 2017; Kielt et al., 2018),
- pain studies (Xu and Sa, 2020),
- sport (Timme and Brand, 2020),
- the influence of negative emotions on driving (Braun et al., 2019), and
- consumer satisfaction from tourism (González-Rodríguez et al., 2020).

**Figure 1:**
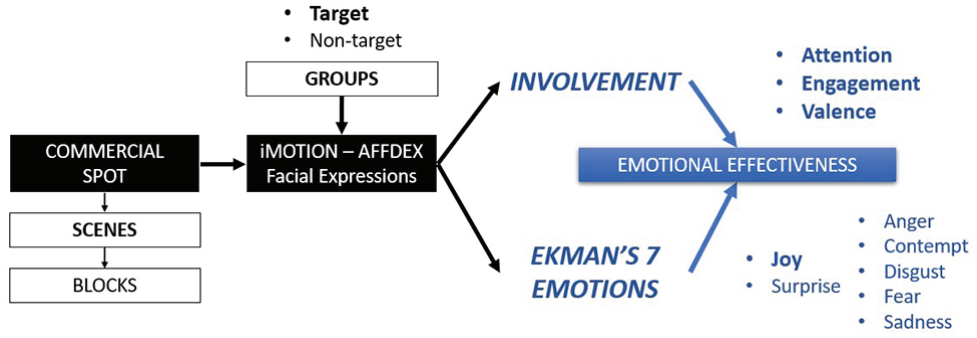
Framework to quantify emotional effectiveness by target group and/or blocks of scenes.

To our knowledge, AFFDEX has not been yet applied in marketing to its full potential. One key novelty of this study is the integration of the three involvement measures together with the analysis of the seven basic emotions to develop a framework to quantify emotional effectiveness of commercial advertising (Figure 1). We believe that the joint use of these complementary measures provides new insights into the emotional response that the advertisement provokes to fully measure the effectiveness of a given spot. Moreover, as will be demonstrated in this article, the differences among scenes of the spots or among the groups of viewers of the advertisement might be analyzed in detail.

The theoretical objective of this research is therefore to shed new light on the quantification of the emotional effectiveness of advertising among different groups based on the measurement and joint specification of emotions and emotional involvement using the analysis of facial expressions provided by AFFDEX and its 10 indicators.

Accordingly, we propose within this framework a set of three hypotheses to measure the emotional effectiveness of advertisement. The first hypothesis is stated as:

- *Hypothesis* 1: An advertisement is emotionally effective whenever
  - the average level of attention is high (≥50 in AFFDEX),
  - the average level of engagement is high (≥50 in AFFDEX),
  - the average level of valence is positive (≥0 in AFFDEX), and
  - the predominant emotion is joy and its average level is high (≥50 in AFFDEX).

Indeed, one of the objectives in advertising communication is to achieve high levels of attention and engagement because emotions are highly correlated with decision-making and memory (Le Blanc et al., 2014). Thus, high levels of engagement to an advertisement influence long-term memory and increase consumer purchase (Loewenstein, 1966; Kiehl et al., 2001; Öhman and Mineka, 2001; Algom et al., 2004; Estes and Verges, 2008; Milosavlejevic and Cerf, 2008; Ramsøy et al., 2012; Le Blanc et al., 2014).

We propose valence to understand the general emotional experience. The analysis of valence allows us to understand the quality, positive or negative, of the emotions. A positive valence in commercial advertising reflects approaching behavior, whereas a negative valence is a sign of distancing behavior (Timme and Brand, 2020). Similarly, in order to demonstrate ad liking, out of the seven emotions, the predominant one must be joy (Tomkovick et al., 2001; Lewinski et al., 2014; Shehu et al., 2016).

A second hypothesis relates to the sequence of scenes or maybe block of scenes of the advertisement and the aim to measure if the emotions are stable throughout the whole length of the spot or some scenes trigger certain emotions (Dimberg et al., 2011).

- *Hypothesis* 2: An advertisement is emotionally effective across scenes whenever the indicators for some scenes are higher on average than the indicators for the rest of the scenes in the spot.

We propose Hypothesis 2 after realizing that spots are usually broken into scenes to compare among spots that differ only in one scene or to focus on the scene of a single spot that triggers the emotions that are sought (Lienhart et al., 1997; Teixeira et al., 2012, 2014; Braun et al., 2013; Vecchiato et al., 2014; Yang et al., 2015). In fact, if Hypothesis 2 is accepted because of differences between scenes, a deeper analysis could and should be carried out to determine why some scenes are more emotional than others. If Hypothesis 2 is rejected, the conclusion might be that the scenes provoke stable emotions throughout the length of the spot.

Finally, an effective advertisement is also one that reaches its target audience and positively influences the emotional attitude and responses of the consumers (Meyers-Levy and Malaviya, 1999; Lee et al., 2015). The experimental objective of many studies is to evaluate the effectiveness of the advertisement in terms of ad liking for the different groups of participants, differentiating between the target group and the rest of participants (Kotler et al., 2000; Ansari and Riasi, 2016; Gountas et al., 2019). The third hypothesis might accordingly be stated as follows:

- *Hypothesis* 3: An advertisement is emotionally effective for the target group whenever the indicators for this group are higher on average than the indicators for the rest of participants.

As a test case to validate the framework, this study aims to quantify the effectiveness of advertisement using a 91-s commercial spot composed by 31 scenes made by Scotch-Brite and its line of scouring pads to celebrate the sixtieth anniversary of the brand’s presence in Spain. The spot shows scenes along the lifetime of women while the study focuses on mature women and moms as the target group to compensate their lifelong efforts on raising children and creating family ties while buying and using its products and developing the brand’s name.

The article is structured as follows. After setting the theoretical background and the framework to measure effectiveness of advertisement in this introduction, “Materials and Methods” section explains the method of analysis based on facial expressions as well as the experimental setting, including the spot and its scenes and the grouping of subjects. “Results” section shows the results by scene and gender and statistically test the hypothesis. “Discussion” section is used to discuss the possibilities of emotions being the tool for marketing in the future.

## Materials and Methods

### AFFDEX, the Analysis of Facial Expressions and Emotional Reactions

Facial expressions are a gateway to the human mind, emotion, and identity. They are a way of relating to others, of sharing understanding and compassion. They are also a way of expressing joy, pain, sorrow, remorse, and lack of understanding (Taggart et al., 2016). These characteristics can be crucial while capturing the key features of a stimulus in the form of a video or image frame. Individual facial recording while watching the computer screen is compared with a biometric database that represents “true” emotional faces, while looking for similarities or even a possible match. Therefore, facial recognition is used to measure and analyze the emotional reactions of the subjects to a given stimulus.

To carry out the emotional measurements in this study, a software platform for biometric measurements research called iMotions was used (iMotions, 2020). This company indicates that its software can combine “eye tracking, facial expression analysis, EEG, GSR, EMG, ECG, and surveys” (Taggart et al., 2016). The platform is used for various types of academic and business research. Version 7.0 was used in this research.

The software records several raw indicators per frame based on biometric measurements or action units while an experimental subject is watching a stimulus on the computer screen: 34 core facial landmarks (jaw, brows, nose…), interocular distance, and head position (yaw, pitch, and roll).

The recorded values for the raw indicators are then transformed by the software underlying models into Ekman’s seven basic emotions. An indicator for each emotion is provided based on the probability of appearance of the emotion, so the range of values for each of them is from 0 to 100. A value of 50 is proposed by AFFDEX as an initial threshold to determine if an emotional response has been detected.

Three involvement indicators are also calculated after combining the raw values. Attention is calculated from the head position and gives an indication of the focus of the individual. Attention ranges from 0 to 100, although is not a probability. Engagement or the level of responsiveness has also a range from 0 to 100. Finally, the range of valence is from −100 to 100, providing an indication of positive, neutral, or negative experience. The initial thresholds are usually arbitrarily set at ±50.

### The Stimulus: The Spot

The stimulus was a spot that belonged to a campaign to mark the sixtieth anniversary of the Scotch-Brite brand’s presence in Spain. The video presentation of the spot lasted 91 s and was broadcast on social networks^1^.

The content of the video describes the accompanying role that a mother plays throughout the life of a child, from birth to adulthood. The video was split into 31 scenes by the advertising company (Table 1). There are 22 real images with family connotations, six frames with text, a black scene, the logo of the sixtieth anniversary, and the campaign’s hashtag.

**Table 1:** Description of the scenes.

Scene number	Content
1	Pregnancy
**2**	**Text: It all started with her**
3	Mother and baby’s hands
4	Taking baby pictures
5	Old family video I
**6**	**Text: For making the impossible possible**
7	Scrubbing with a hanging baby
8	Baby bathing
9	Girl on a mop
**10**	**Text: She taught us how to grow**
11	Child in arms over the horizon
12	Brushing teeth as a family
13	Learning how to ride a bike
**14**	**Text: She gave us the most sincere love**
15	Hug girl-mother
16	Family in the country
17	Girl with a thermometer
**18**	**Text: Sharing the best moments with us**
19	Girl making soap bubbles
20	Old family video II
21	Family together
**22**	**Text: Because a mother shines with her own light**
23	Interaction between grandmother and granddaughter
24	Interaction between mother and daughter on her wedding day
25	Hug between three women
26	Family reunited with grandma
27	Mother and Child
28	Two women making a heart with their hands
29	Fade to black
**30**	**Brand logo on the 60th anniversary**
**31**	**Text**: #I cannot be without her

### The Experiment

Recruitment to watch the spot was done through a snowball process. Snowball is traditionally used whenever the theme of study is relatively new (Strydom and Delport, 2005) and for which it is difficult to find participants (Babbie, 2008). This type of sampling is particularly used to influence in the buying process of both consumers and nonconsumers (Rozalia 2007, Venter et al., 2011).

The snowball process helps complete the sample based on a specified set of criteria (Henning et al., 2004), once the first subjects are selected (McDaniel and Gates, 2007). For this research, the target group had to be habitual consumers and users of scoring pads and older than 18 years. The first requisite, that of consumers and users, drove the sample toward women, because they are primarily those that do the housework. Seventy-eight percent of European women (84.5% in Spain) perform house cleaning (which includes dishwashing), whereas only 33.7% (41.9% in Spain) of the men do (EIGE, 2018).

The recruiting process started with a first sample provided by the company that produced the spot, both consumers and nonconsumers. The first contact was made by telephone after randomly selecting the potential participants, and if available, they were scheduled to go to the research site and participate by watching the video. These selected subjects were also asked to provide contacts to guarantee a continuous chain of sampling (Strydom and Delport, 2005).

The subjects were divided into two large groups: that of product consumers or the target group (mature women between the ages of 50 and 65 years) and that of nonconsumers. To further divide the nonconsumers, while keeping the gender perspective, the decision was to divide the women in two groups by age (young and middle aged) and assign men to a third group. Four groups were therefore available at the initial stages of sampling: three for women and one for men.

However, while interviewing the initial set of participants, we identified some rejection or repulse to the theme under study, that of washing dishes with scouring pads. As a consequence, it made sense to include women with a strong feminist sensitivity as a separate fifth group. This additional “feminist” group was identified by asking women the following question:

“As for the social movements that claim to incorporate the gender perspective in the different instances of society, up to what level do you identify with this type of proposal?”

The answer had to be provided using a 10-point Likert scale, ranging from 1 (nothing identified) to 10 (fully identified). Those women regardless of age who responded with scores between 8 and 10 were included in this special group.

As a result of the snowball process, 100 people participated in the experiment (80 women and 20 men, between 18 and 65 years old). The five groups with 20 people each were then defined as follows:

- group 1: young women, aged 18–29 years
- group 2: middle-aged women, aged 30–49 years
- group 3: mature women, aged 50–65 years
- group 4: women with a strong feminist sensitivity, aged 18–65 years
- group 5: men, aged 35–65 years

The experiment was carried out at the Brain Research Lab of the Universidad Rey Juan Carlos, Madrid, Spain, between November 10, 2018, and December 10, 2018. The room was kept at a constant temperature of 22°C throughout the experimentation phase. The room was isolated from the outside by means of a soundproofing system. We also used the same indirect lighting system for all the participants, so the emotional comparison across groups of subjects was robust.

The participants were scheduled in 10-min intervals and viewed the video on their own. The subjects entered the room, where they sat in front of a 14-inch monitor on which the spot would be projected, at a distance of 50 cm from the screen. On top the screen, there was a Logitech HD recording camera. iMotions was then calibrated to ensure that the facial microexpression detection mask captured the entire face of the subjects. Once an adjustment of 96% was achieved by iMotions, the spot was viewed. They did not know *a priori* what the spot was about.

The participants received a compensation of 20 euros for their collaboration. All subjects signed *a priori* a consent form that considers all aspects of data protection. This consent ensures the nonidentification of the participants, nor the dissemination of individualized data of a personal nature.

### Statistical Methods

We developed a database with the 10 indicators provided by AFFDEX by scene and subject. Because 100 individuals watched the 31 scenes of spot, there were 3,100 registers in the database, each with 10 columns, one per indicator. Each indicator ranges between 0 and 100, indicating the likelihood of the emotion being present (0 = absent, 100 = present). Therefore, the higher the values that are recorded, the higher the emotional levels that are shown. Two additional columns were added to identify each register with the levels of the two control variables: scene (1–31) and group (1–5).

A descriptive analysis was first carried out to obtain an overall idea of the emotional responsiveness to the spot. Besides providing plots and summary statistics of the whole set of records, the extreme values, defined as those above the 90^th^ percentile and below the 10^th^ percentile, were highlighted in colors (green for high, red for low).

An inferential analysis was then undertaken to compare the average values of the different levels of the control variables. The general linear analysis of variance (ANOVA) model was used to capture the differences among levels of one variable at a time, as well as both variables together. A series of *F* tests (one per each of the 10 emotional indicators) were used to statistically reject (or not) the null hypothesis of equality of means across levels. A significance value of 5% was used as the threshold for rejection. The corresponding values of *p* were calculated, highlighting those that are lower than 5%, to demonstrate which variables significantly influence on the emotional responses by group and/or scene.

Concerning which levels of the variable are significantly different than the rest, we performed a series of *t*-tests, maintaining the significance level at 5% The values of *p* are provided, highlighting those that are lower than 5%, to demonstrate which levels of the variables significantly influence on the emotions. A “+” sign was used to demonstrate higher values than average, and a “−” to depict lower values than average.

## Results

### Overall Results

**Figure 2:**
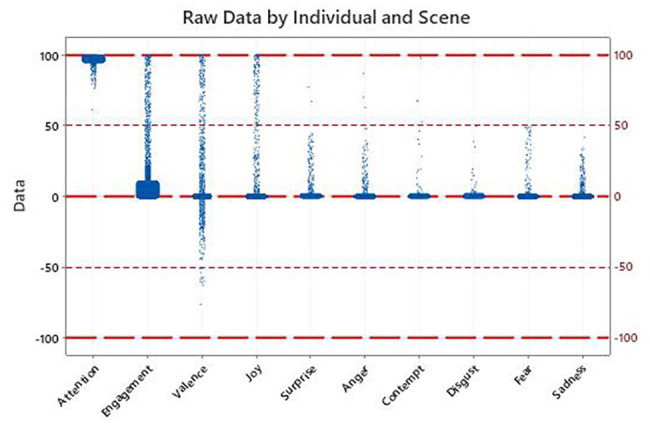
Descriptive plot for the whole sample by emotional response.

The results for the three involvement indicators and the seven basic emotions are shown in Table 2 and depicted in Figure 2. Each observation in the plot is the average measurement by individual and scene, for a total of 3,100 samples (31 scenes and 100 subjects) in each of the 10 plots. The lower and upper sides of the boxes correspond to the first and third quartiles, with the dots indicating the values outside the box. For illustrative purposes, and following AFFDEX initial configuration, thresholds are shown at ±50. Nevertheless, the research is based on the whole set of values.

**Table 2:** Descriptive statistics for the whole sample by emotional response.

Variable	N	Mean	SE Mean	StDev	Minimum	Q1	Median	Q3	Maximum	Above 50 (%)
Attention	3,100	96.75	0.04	2.50	61.22	96.06	97.54	98.32	98.72	100
Engagement	3,100	12.34	0.45	25.22	0.05	0.09	0.19	9.27	99.92	10
Valence	3,100	4.44	0.38	20.90	−76.24	0.00	0.00	0.00	99.94	5.4
Joy	3,100	4.82	0.33	18.35	0.00	0.00	0.00	0.00	99.88	4.7
Surprise	3,100	1.12	0.08	4.68	0.00	0.19	0.20	0.27	77.20	0.1
Anger	3,100	0.70	0.08	4.27	0.00	0.00	0.00	0.01	86.73	0.1
Contempt	3,100	0.42	0.06	3.51	0.00	0.18	0.19	0.19	98.71	0.2
Disgust	3,100	0.48	0.03	1.48	0.00	0.28	0.42	0.43	49.63	0
Fear	3,100	0.56	0.07	4.13	0.00	0.00	0.00	0.01	49.15	0
Sadness	3,100	0.64	0.06	3.10	0.00	0.01	0.02	0.03	41.74	0

Only four of the indicators show reads consistently outside the thresholds and almost reaching the maximum value of 100: attention, engagement, valence, and joy. None of the five negative emotions reach 100, with only a few combinations of scene and individual above 50 for anger (three reads, 0.1%) and contempt (five reads, 0.02%). Moreover, three of the negative emotions (disgust, fear, and sadness) do not even reach the initial threshold.

Starting with attention, all the values are above 75 except for only a single value at 61.22. The lower quartile is above 96. Therefore, attention during the spot is kept across the individuals. High mean values (≥50) for this indicator show involvement and therefore emotional effectiveness, corroborating Hypothesis 1a.

Engagement shows most of the values under the threshold, but 309 of the 3,100 (10%) are above the limit. Whereas the third quartile is just at 9.27 and the average at 12.34. Because these values are not high (<50), Hypothesis 1b is not fully supported for the entire sample, but it looks worthy to investigate emotional reactions further by segregating by scene and group.

The same happens for valence, which shows more values above the positive threshold (168, 5.4%, maximum at 99.94) than below −50 (12, 0.4%, minimum at −76.24). Hypothesis 1c is not fully supported either for the entire sample, because not a significant amount of the samples is positive.

The predominant emotion is joy, with the percentage of values above 50 (probability of the emotion being present) being 4.7% (147 observations). Once again, from a descriptive perspective, the percentage is low enough to not accept Hypothesis 1d for the entire sample.

The overall descriptive analysis implies that the positive emotions are present at times, whereas the negative ones are almost never shown. Although the hypotheses are not fully accepted for the whole sample, the aim behind the spot is that the positive emotions are shown primarily by the target group, triggered by the sequence of images and text. Let us proceed therefore with the inferential analysis by scene and gender and age groups in order to quantify emotional effectiveness.

### Results by Scene

**Figure 3:**
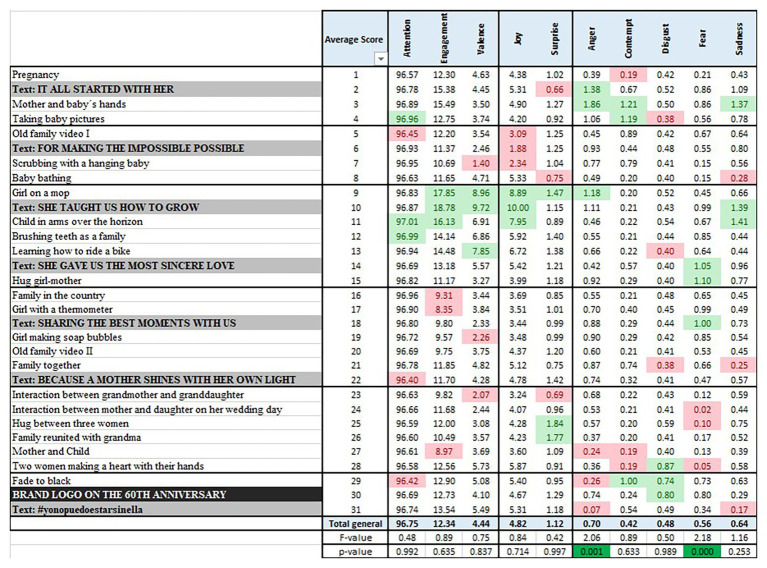
Inferential analysis per scene.

Figure 3 shows the results averaged across individuals for each of the 31 scenes. For each of the 10 emotional indicators, the three scenes (first decile or top 10%) with the highest values are highlighted in green, and the three scenes with the lowest values in red.

The main green zone for positive emotions and involvement indicators goes from scene 9 to scene 15. Attention provides the two highest values in this block of scenes, with engagement, valence, and joy providing all of their top three scores. Fear and sadness also give maxima in this block. The text scenes are “She taught us how to grow” and “She gave us the most sincere love,” both related to the important role of moms. It must be remembered at this point that mature women are the target of the spot.

The negative emotions are higher at the beginning (scenes 1–4, anger and contempt) and at the end of the spot (29–31, disgust). It is eye catching that the scene with the company logo, scene 30, shows a peak of disgust, not showing any high values for the rest of the indicators except for surprise. The lower peaks of involvement (in red) are found between scenes 16 and 22, whereas joy is low between scenes 5 and 8.

To statistically test if these visual differences are significant per indicator, a series of one-way ANOVA test are performed. The null hypothesis is that the average value for each scene is the same, and the alternative hypothesis is that not all the scene averages are equal. The bottom rows of Figure 2 include the values of *F* and the *p* of each test (*p* < 0.05 for rejection). Only anger (three individual values above 50) and fear (0 observations over 50), both negative emotions with very low averages, show differences across scenes. Their peaks surprisingly occur while positive emotions flourish, whereas their valleys are found at the end of the spot.

The summary is that Hypothesis 2 is not corroborated for scenes because no significant differences are found across them. Therefore, no clear emotional effectiveness by scene is shown. It is worth highlighting that the three involvement indicators and joy (relating all four to Hypothesis 1) are stable throughout.

To deeper analyze the spot, we continue the study in terms of block of scenes and not just single scenes. After the results of the descriptive analysis based on top and bottom deciles, we have broken the scenes into five blocks, which have been named according to a common theme:

- block 1: birth (1–4),
- block 2: first cares (5–8),
- block 3: teaching growth and love (9–15),
- block 4: sharing the best moments (16–22),
- block 5: reunion of three generations (23–28), and
- block 6: logo (29–31).

**Figure 4:**
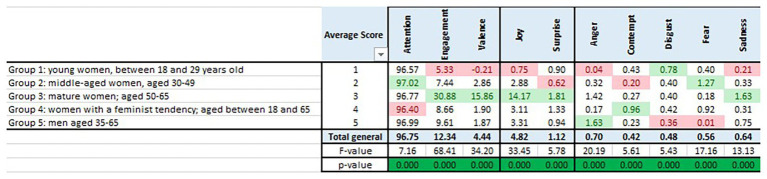
Inferential analysis per group.

Figure 4 includes the statistical analysis by block. Differences are found on average emotional involvement across blocks of scenes in engagement and valence, with block 3 related to growth and love obtaining the highest results for involvement and positive emotions. Correspondingly, block 3 causes joy, although it is also significantly different in fear.

Block 1, related to birth, is significantly high in negative emotions of contempt and sadness. Block 2 shows low values for positive emotions, block 4 for engagement, and block 5 for attention and contempt. Finally, and although the indicator is not significant, block 6 shows a peak of contempt.

Therefore, Hypothesis 2 is corroborated for blocks, providing an indication that the advertisement provokes emotions unevenly along the duration of the commercial. The spot is emotionally effective for blocks of scenes, in this case, generating higher positive involvement and joy for block 3: teaching growth and love.

### Results by Group

**Figure 5:**
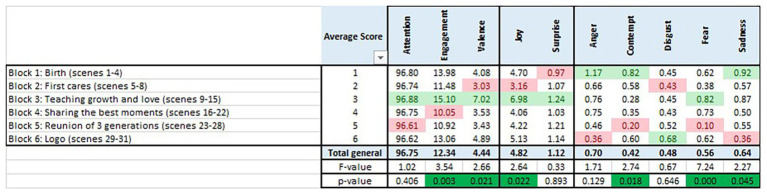
Inferential analysis per block.

The analysis of the groups is critical also to determine ad liking, specifically for the target group. Figure 5 has the same format as Figures 3, 4 but, instead of segregating by scene, the averages are calculated for each of the five groups in which the individuals were pooled together. The maximum values per indicator are highlighted in green, and the minimum values in red.

Indeed, all groups behave differently, as demonstrated by the one-way ANOVA shown at the bottom row of Figure 5. Differences across groups in terms of average emotional response are found significant for each and every one of the 10 indicators. All the values of *p* are 0, indicating that the null hypothesis of equality of averages across groups is rejected in favor of the alternative hypothesis of differences in averages across groups.

Moreover, group 3 of mature women gets the top values in engagement, valence, joy, and surprise, and the middle one in attention (although at a very high 96.77). Therefore, for this group, which is the target group, all four indicators included in Hypothesis 1 are higher than those of the nontarget groups.

Compared to the average behavior, each group shows peaks of negative emotions, different in each case. Group 1 of young women demonstrates negative valence and disgust. Group 2 of middle-aged women shows attention but no emotions other than some fear. Group 3 of mature women exhibit sadness. Group 4 lacks attention while showing some contempt. Group 5 demonstrates some anger.

The summary of this section is consequently that Hypothesis 3 is corroborated. The spot is effective because involvement and joy are higher for the target group, in this case, the mature women.

### Results by Group and Block of Scenes

We finalize the analysis by performing a two-way ANOVA to jointly study scenes (or blocks) and groups. As expected, following the rationale and conclusions of the previous sections, group is significant across indicators (*p* = 0), and scene is nonsignificant in all the cases (*p* ≥ 0.277), so it looks more appropriate to direct the efforts toward studying the relationship between groups and blocks of scenes.

**Figure 6:**
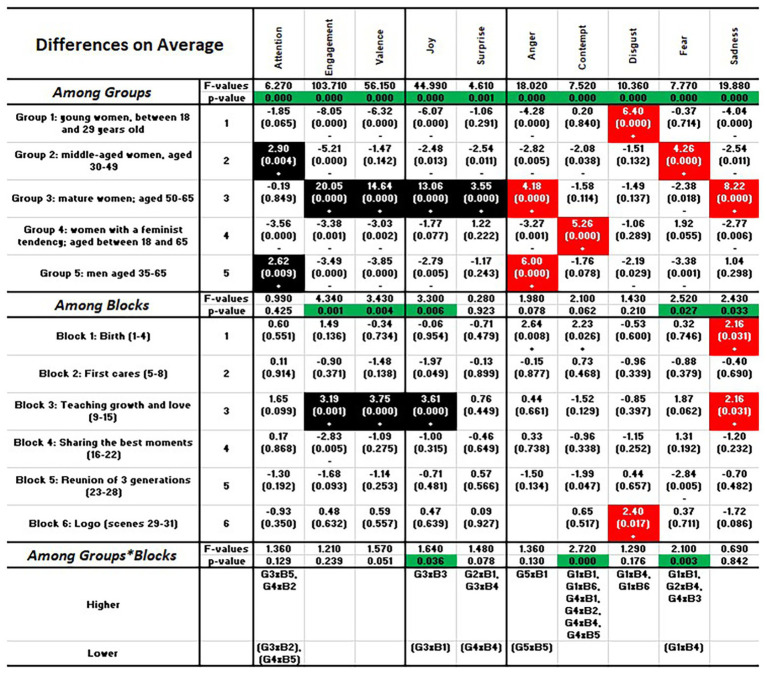
Inferential analysis per group and block.

Figure 6 shows the two-way ANOVA by group and block of scenes that helps summarize the whole study. The first part of the table shows the analysis of significance for the variable “group,” the second for the variable “block,” and the third for their interaction “group × block.” The first row of each section includes the *F* values, and the second the values of *p* for each indicator. For the variable group, there are significant differences on average for each of the 10 indicators (*p* < 0.05, highlighted in green). For block, there are differences for engagement, valence, and joy, and also for fear and sadness. For the interaction, differences are found for joy, contempt, and fear.

It is worth mentioning at this point that, except for two values of *p* that are close to 1, the rest of the values of *p* are below 0.25, a threshold that is commonly used for exploratory purposes. Accordingly, we have analyzed all the indicators while highlighting the values for group, block, or the interaction that are responsible for determining the significant differences. These differences are depicted with a “+” (if the value is significantly higher than average) or a “−” sign (if significantly lower) in the ANOVA table. For example, for group 2, the “+” sign in the attention column indicates that this group has paid significantly more attention than the average attention for all the groups. The “−” sign in the Engagement column indicates that group 2 was significantly less engaged than the average.

For groups, and concerning positive emotions, group 3 of mature women is the only group above average, the exception being attention, in which group 2 of middle-aged women and group 5 of men excel. For negative emotions, group 1 of young women is above average in disgust, group 2 of middle-aged women in fear, group 3 of mature women in anger and sadness, group 4 of feminists in contempt and group 5 of men in anger, corroborating the importance of the “group” variable, as anticipated by the one-way study.

For blocks, block 1, related to birth, causes higher responses in negative emotions (anger, contempt, and surprise); block 3, with the focus on the mom, shows higher responses in engagement, valence, joy, and sadness; and block 6, the logo and the hashtag, provokes disgust.

For the group × block interaction, we list those specific interactions that are significantly “higher” than average and those that are significantly “lower,” the latter within parenthesis. For example, and with respect to attention, two combinations are higher than average: group 3 × block 5 (the interaction between mature women and the reunion of generations) and group 4 × block 2 (feminists and first cares). Two others are lower than average: group 3 × block 2 (mature women and first cares) and group 4 and block 5 (feminists and reunion of three generations).

### Assessment of the Spot

The results indicate that the spot is indeed emotionally effective because it generates involvement and positive emotions (Hypothesis 1) on the target group, indicators that are statistically higher than the average for the rest of the groups (Hypothesis 3). Mature women like the spot especially whenever the role of the mother is stressed, even showing some sadness, probably due to remembrance of old times.

The rejection of Hypothesis 2 in block of scenes indicates that ad liking might be improved especially for the nontarget group, addressing the scenes or blocks of scenes that cause negative emotions. By looking at the interactions that generate “higher” averages for the negative emotions, spot designers might understand better the “brain” of the participants and the groups they represent. It is eye-striking that each group is characterized by one negative emotion, which is usually triggered by a few blocks of the spot.

Young females (group 1) show disgust, the opposite of ad liking. This emotion is caused by blocks B4 (sharing best moments) and B6 (logo). The explanation might be that these youngsters do not like the best moments with mom or in family. It is worth to remember that they probably have not had relation with the advertised brand.

Middle-age females (group 2) show fear, as well as attention. They like the ad, but emotions are caused by block B4 (sharing best moments). The interpretation is that they are feeling mixed emotions about their life experiences.

Feminists (group 4) show contempt throughout the spot. All interactions between blocks of scenes and disgust are significantly high, except for block B4, which provokes low surprise. Feminist might not like family traditions or pregnancy periods.

Men (group 5) show anger, as well as attention. After showing anger during the first block, their attention level is high, indicating ad liking but without showing positive emotions. It is like they are watching from the outside and not showing extraordinary emotional responses.

The targeted mature women (group 3) show anger and sadness, although not particularly caused by any block of scenes. These two emotions might not be “negative” for this spot in particular as they must be related to reminiscences of the past. In fact, those negative emotions are justified in the literature as an empathy mediator (Eisenberg et al., 1994; Sonnby-Borgström, 2002; Dimberg et al., 2011; Johnson et al., 2014; Richaud and Mesurado, 2016; Rawal and Saavedra, 2017). Anyhow, group 3 however shows above-average involvement and joy.

After this analysis by groups, we can conclude that emotions as expected are a good predictor of ad liking, which is the key measure of emotional effectiveness of advertisement. The combinations of positive and negative emotions that have been found in each of the groups clearly define the groups, relating them to their stage in life. As a conclusion, marketing professionals therefore have tools to measure emotional effectiveness of advertising before and during campaigns.

## Discussion

One necessary approach in this day and age to quantify the effectiveness of advertisements is to resort to emotions and emotional responses in the quest for properly measuring “ad liking and purchase intent” (McDuff et al., 2015). We have proposed a framework composed of a set of three hypotheses to determine the emotional effectiveness of spots and blocks of scenes primarily focusing on ad liking. The novelty was to merge emotional responses, covering the seven basic emotions of Ekman, with involvement, including attention engagement and valence. Under this emotional perspective, the spot must cause involvement and joy in high, constant levels throughout the scenes for the target group.

We have tested the framework with a commercial spot that strived for strong positive emotional reactions among the target group. The combined use of involvement and emotional indicators has proven to be more than satisfactory. There is a direct relationship between blocks and experimental groups, indicating that the emotional responses of the different groups vary with the composition of the groups. On that regard, if the spot was supposed to be attractive to several groups, the effectiveness is lost outside the target group (Kotler et al., 2000). The target group is emotionally more attracted to the spot. The results after analyzing a spot of scouring pads help validate the framework and determine that unconscious emotional responses are liable to be used to quantify effectiveness of advertisement. Therefore, the proposed framework should and must be used also before a spot is marketed to increase its emotional effectiveness.

Several limitations or words of caution might be mentioned at this point. The first one relates to the application of the proposed framework to any sort of spots. The framework looks to be readily usable for advertisements whose aim is to provoke positive emotions throughout the length of the spot. That is usually the case of commercial spots, although some also try to provoke peaks of emotions at certain (blocks of) scenes of the spot. The framework might be used to test these “peaky” commercials, because the null hypothesis of Hypothesis 2 is stability, and therefore the alternative hypothesis is lack of stability. In fact, for the test case that was used for this research, an analysis by scene and by block provided different results. No differences were found among scenes, but differences were found among blocks. We argue that the important feature of the framework is to be able to highlight differences, whether that is a proof of effectiveness or lack of it.

The spots might also look for negative emotions (Rivera et al., 2000). Adding (or substituting) emotions to Hypothesis 1 is straightforward. The methods of analysis of emotions other than joy are the same. In fact, we have analyzed in this research all of the seven Ekman’s emotions, although we have focused primarily on joy because, to demonstrate ad liking, out of the seven emotions, the predominant one must be joy (Tomkovick et al., 2001; Lewinski et al., 2014; Shehu et al., 2016). We, however, acknowledge that there are certain situations in which responses based on negative emotions are sought for example, for fear (Basil et al., 2008).

There are also several directions for future improvement and research. To further quantity purchase intent and recall, questionnaires might be added to spot viewing. Moreover, the questionnaires might be shown to the participants on the screen, so emotions might be measured both while watching the spot and while filling the questionnaire. An additional step is to measure empathy, which in neuroscience is usually quantified with fMRI and EEG (Mouras et al., 2008; Neumann and Westbury, 2011; Touchette and Lee, 2016), while measuring the reaction of mirror neurons to the stimuli; this approach, however, could make the research unaffordable for a reasonable sample size.

Continuing with technology, while the framework is based on neuroscience techniques and tools, namely, those provided by the software platform AFFDEX, other software that quantify the proposed indicators included in the hypotheses might be also used. The state-of-the-art platform AFFDEX translates microfacial expressions at the same time into attention, engagement, valence, and the seven basic emotions of Ekman. Many steps forward are, however, necessary to be able to improve the study of the consumers’ brain using these tools. We have only used the analysis of facial microexpressions, but some other techniques might be simultaneously used, for example, eye tracking or EEG. The statistical analysis of this type of combined output will shed new light on how the emotions are trigged, in lieu of a more thorough analysis of emotional effectiveness of advertising.

To conclude, we have been able to demonstrate that unconscious emotional responses might be used to understand more about the consumers’ brain. In the era of big data and the internet of things, the more indicators are present, the better the analysis might be. Consistently measuring emotions based on the principles of consumer neuroscience might be the key to understanding consumer behavior and effectiveness of advertisement in the upcoming years.

## Data Availability Statement

The raw data supporting the conclusions of this article will be made available by the authors, without undue reservation.

## Ethics Statement

The studies involving human participants were reviewed and approved by Ethics Committee – 3M Spain SL. The patients/participants provided their written informed consent to participate in this study.

## Author Contributions

FO and DS designed the study. DS acquired the data. FO prepared and analyzed the data. FO and DS drafted the manuscript. All authors revised and approved the final version of the manuscript.

### Conflict of Interest

The authors declare that the research was conducted in the absence of any commercial or financial relationships that could be construed as a potential conflict of interest.

## Footnotes

^1^The video is available at https://www.youtube.com/watch?v=yaLqgWmoh2Y
